# Comparison of Visceral Fat Measures with Cardiometabolic Risk Factors in Healthy Adults

**DOI:** 10.1371/journal.pone.0153031

**Published:** 2016-04-04

**Authors:** Kyoungjune Pak, Seung Hun Lee, Jeong Gyu Lee, Ju Won Seok, In Joo Kim

**Affiliations:** 1 Department of Nuclear Medicine and Biomedical Research Institute, Pusan National University Hospital, Busan, Korea; 2 Department of Family Medicine and Biomedical Research Institute, Pusan National University Hospital, Busan, Korea; 3 Department of Nuclear Medicine, Chung-Ang University College of Medicine, Seoul, Korea; The Ohio State University, UNITED STATES

## Abstract

We aimed to evaluate the associations of visceral adiposity with cardiometabolic risk factors in normal subjects with integrated ^18^F-Fluorodeoxyglucose (FDG) positron emission tomography (PET)/computed tomography (CT). A total of 58 normal subjects who underwent ^18^F-FDG PET/CT scan for cancer screening were included in this study. Volume and average Hounsfield unit (HU) of visceral adipose tissue (VAT) was measured from CT components of integrated PET/CT. Standardized uptake values (SUVmax) of liver, spleen, lumbar spine and ascending aorta (AA) were measured from PET components of integrated PET/CT. Body mass index (coefficient 78.25, p = 0.0259), glucose (37.62, p<0.0001), insulin (348.90, p = 0.0011), logarithmic transformation of homeostatic model assessment index-insulin resistance (-2118.37, p = 0.0007), and VAT HU (-134.99, p<0.0001) were independently associated with VAT volume. Glucose (0.1187, p = 0.0098) and VAT volume (-0.004, p<0.0001) were found to be associated with VAT HU. Both VAT volume and VAT HU of whole abdominal cavity is significantly associated with cardiometabolic risk factors.

## Introduction

According to the most recent estimates of the 2013 Korean national health and nutrition examination survey, 37.8% or men and 25.1% of women classified as obese[1]. The prevalence of obesity has increased over the last decade and numerous prospective cohort studies have shown that obese persons, defined by a body mass index (BMI), have an increased risk of hypertension, cardiovascular disease, and all cause mortality [1–4]. Furthermore, the distribution of fat depots, such as visceral abdominal adipose tissue (VAT), independent of overall obesity, has been associated with cardiometabolic risks [5, 6].

Several studies have shown the area of VAT, measured by single-slice computed tomography (CT) image at L4-5 intervertebral space, could reflect the amount of VAT and may be the predictor of cardiometabolic complications [7, 8]. However, using a single slice of CT image to represent total VAT may not be appropriate for all population because of there are race, sex or age differences in the distribution of VAT across the abdomen [9]. Furthermore, controversy exists which measurement site is the best correlated to not only VAT volumes but also cardiometabolic risk factors [9–11]. Thus, volumetric quantification of whole abdomen is the gold standard to assess amount of VAT [5, 12].

Previous studies suggested CT attenuation of adipose tissue, measured in Hounsfield Units (HU) as a density on x-ray imaging was adopted as a marker of fat quality and lower CT attenuation was associated with higher cardiovascular disease risk, independent of fat volume [13–15]. Although, these studies demonstrated that regional CT imaging allows the assessment of volume and density of adipose tissue, they could not assessed the whole adipose tissue of abdominal cavity.

In the present study, we measured total volume and attenuation of whole VAT of abdominal cavity with integrated ^18^F-fluorodeoxyglucose (FDG) positron emission tomography (PET)/CT scans to evaluate the associations between cardiometabolic risks and other variables from PET/CT.

## Materials and Methods

### Patients

**Fig 1:**
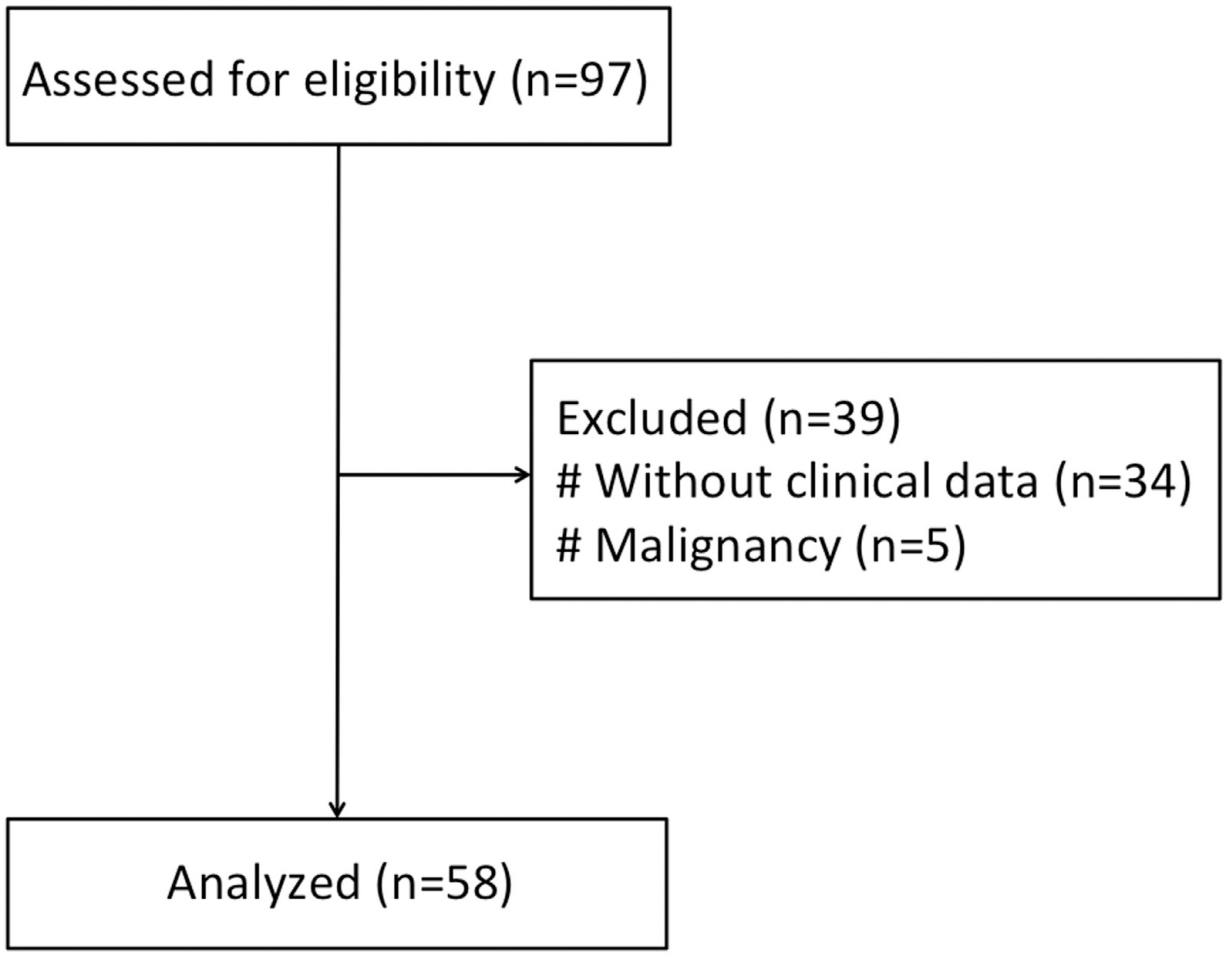
Flow diagram of study subjects.

A total of 58 subjects who underwent ^18^F-FDG PET/CT scan for cancer screening between February 2011 and September 2014 were included in this study. Study participants with known malignancy or age<18 years were excluded (Fig 1).

This study was designed as a retrospective review of participants’ charts. Participants’ information was anonymized by JG Lee. The consent from participants was not needed according to Institutional Review Board of Pusan National University Hospital (PNUH-2015-07892).

### Measurement

Body mass index (BMI) was calculated as weight (kg) divided by height squared (m^2^). Waist circumference was measured at the narrowest point between the lower border of the rib cage and the iliac crest, at the end of a normal expiration of breath and to the nearest 0.1cm. Percentage of body fat and total fat mass was measured by bioelectric impedance analysis (Inbody 3.0, Biospace Co, Ltd, Korea). Fasting plasma glucose was measured by the glucose oxidase method using a Synchron LX20 (Beckman Coulter, Fullerton, CA, USA). Fasting insulin was measured using a radioimmunoassay (Diagnostic Product Corporation, Los Angeles, CA, USA) with antibody-coated tubes. A mercury sphygmomanometer was used for measurement of blood pressure of each subject in sitting position after a 10-min rest. Homeostatic model assessment index-insulin resistance (HOMA-IR) was calculated using the formula for estimation of insulin sensitivity: fasting insulin (IU/mL) x fasting glucose (mg/dL)/405[16]. Lipid profile was measured by an enzymatic colorimetric method using with an autoanalyzer (Hitachi 747; Hitachi Corp., Tokyo, Japan).

### Integrated ^18^F-FDG PET/CT

Patients were injected intravenously with 5.18MBq/kg of ^18^F-FDG, following a fasting period of at least 6 h to achieve a blood glucose level of < 140 mg/kg. PET/CT scans commenced 60 min following injection using integrated PET/CT scanners (Gemini TF, Philips, Milpitas, CA, USA). During image acquisition, a CT scan was obtained first for attenuation correction with a slice thickness of 4mm (120kV), and an emission scan was obtained consecutively from the skull base to the proximal thigh with a field-of-view of 576mm. PET images were reconstructed using an iterative algorithm (ordered-subset expectation maximization, iteration: 3, subsets: 33) with an image matrix size of 144 x 144. Two nuclear physicians, blinded to clinical data, reviewed retrospectively PET/CT datasets.

### Image analysis

^18^F-FDG PET/CT images were reviewed by 2 experienced nuclear physicians with pmod version 3.2 (PMOD Technologies Ltd, Zürich, Switzerland).

#### a. PET

Standardized uptake values (SUV) were calculated as the tissue concentration of radioactivity (kBq/mL) divided by the injected dose per weight (kBq/g). A circular region of interest with a diameter of 2cm was placed in the right lobe of the liver (Liver SUV) and center of spleen (Spleen SUV), avoiding vessels, bile ducts, calcifications, and artifacts to measure maximum SUV (SUVmax). SUVmax of each lumbar spine was measured on body of L1-5, was averaged, and defined as lumbar SUV. SUVmax of blood pool was evaluated within the wall of the ascending aorta on every 5 axial image, and the average was defined as AA SUV.

#### b. CT

**Fig 2:**
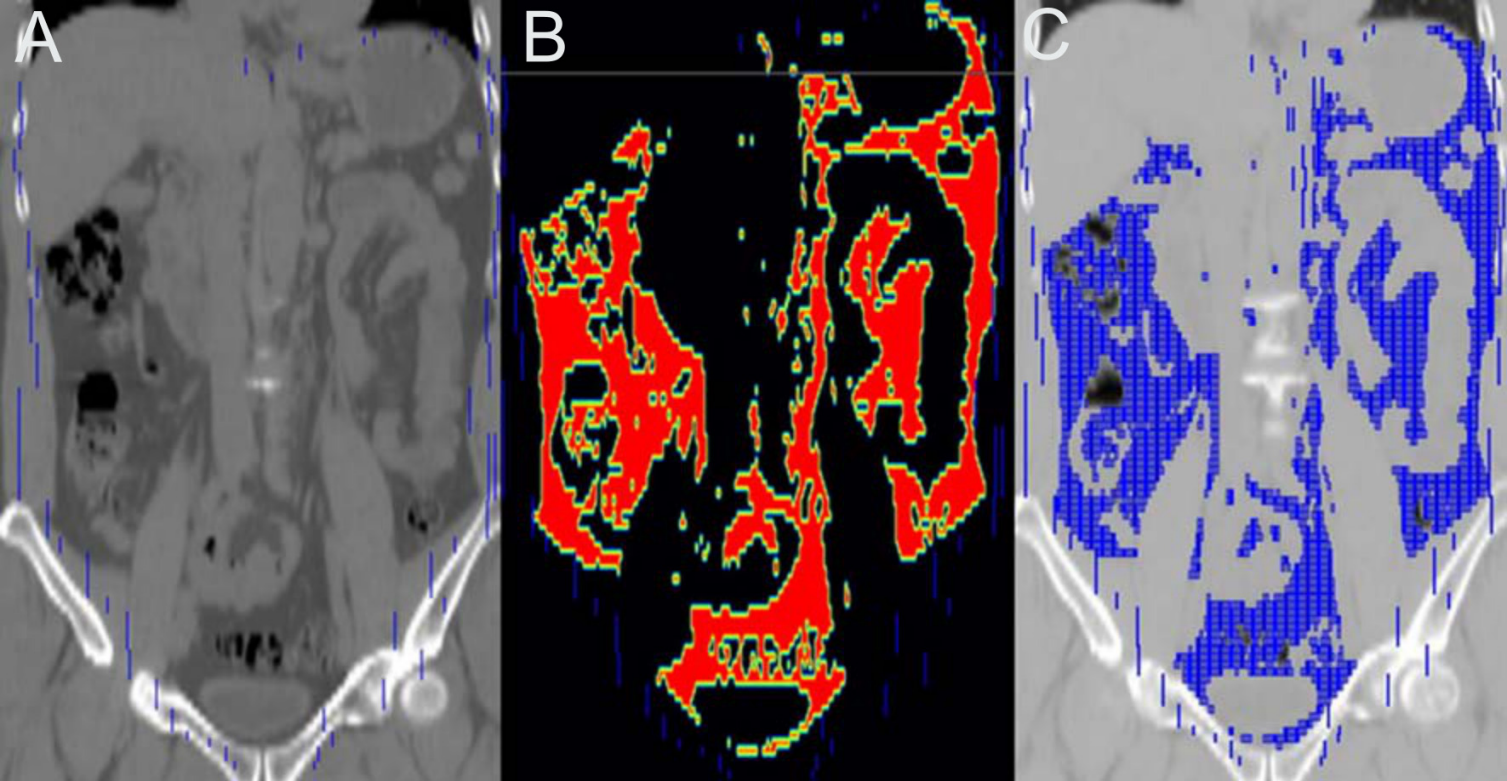
Measurement of whole visceral adipose tissue. (A) Manually outlining volume-of-interest of the abdominal muscular wall, (B) defining voxels between -195HU and -45HU, making a template atlas, and (C) applying a template atlas to CT.

Volume (VAT volume, ml) and average Hounsfield unit (VAT HU) of visceral adipose tissue was measured from CT components of integrated PET/CT scans. VAT was delineated as follows: 1) manually outlining VOI of the abdominal muscular wall excluding vertebral column and paraspinal muscles, 2) defining voxels between -195HU and -45HU, making a template atlas, 4) applying a template atlas to CT, and 5) measuring parameters (Fig 2).

### Statistical analysis

All continuous variables were expressed as mean±standard deviation. Subjects were divided into 2 groups with median value of VAT volume and VAT HU, analyzed with T-test. D’Agostino-Pearson test was adopted to assess normal distribution. Multiple regression method was used to examine the relationship between VAT volume, VAT HU and other variables after adjustment with age and sex. Variables with p value <0.05 were entered into model with enter method. A p value of < 0.05 was considered statistically significant. All statistical analyses were performed using the MedCalc software package (ver. 12.6.0.0, MedCalc, Mariakerke, Belgium).

## Results

Demographics are summarized in Table 1. The mean age was 48.4±8.3 years with a range between 39 and 73. Thirty-eight subjects were male, 20 were female. The mean VAT volume was 3,285ml and the mean VAT HU was -95.2.

**Table 1:** Demographics.

Characteristic	All	High VAT volume	Low VAT volume	p
Age (years)	48.4±8.3	49.2±8.1	47.7±8.5	0.4900
Sex (M/F)	38/20	26/3	12/17	0.0002
BMI (kg/m^2^)	25.4±4.9	26.2±2.0	22.4±2.4	<0.0001
Waist circumference (cm)	84.7±7.6	90.4±4.4	78.9±5.5	<0.0001
AST (IU/L)	21.7±6.6	23.6±6.8	19.7±5.9	0.0251
ALT (IU/L)	22.4±12.3	28.1±12.8	16.7±8.9	0.0002
Hb (g/dL)	14.4±1.5	15.2±1.2	13.7±1.5	0.0001
Hct (%)	42.5±4.0	44.3±3.3	40.7±3.9	0.0004
WBC (x10^3^/uL)	5.7±1.7	6.5±1.8	4.9±1.1	0.0001
Platelet (x10^3^/uL)	235.9±52.2	234.2±62.0	237.6±41.1	0.8077
Lipid				
Total cholesterol (mg/dL)	195.4±33.4	194.2±33.5	196.7±33.9	0.7742
LDL cholesterol (mg/dL)	125.4±31.8	126.8±31.6	124.0±32.4	0.7411
HDL cholesterol (mg/dL)	51.3±14.5	46.0±13.8	56.6±13.5	0.0049
lnTg (mg/dL)	4.8±0.5	4.9±0.5	4.7±0.4	0.1340
Glucose (mg/dL)	91±14	96.0±16.5	86.0±7.4	0.0041
Insulin (uIU/mL)	5.3±2.4	6.2±2.9	4.4±1.2	0.0021
lnHOMA-IR	0.1±0.5	0.3±0.5	-0.1±0.3	0.0012
CRP (mg/dL)	0.1±0.1	0.11±0.10	0.04±0.06	0.0016
AA SUV	2.0±0.3	2.1±0.3	1.8±0.3	0.0006
Lumbar SUV	1.6±0.3	1.7±0.3	1.5±0.2	0.0045
Liver SUV	2.5±0.3	2.6±0.3	2.3±0.2	<0.0001
Spleen SUV	1.9±0.3	2.0±0.3	1.7±0.3	<0.0001
VAT HU	-95.2±5.6	-99.1±4.0	-91.4±4.2	<0.0001

VAT, visceral adipose tissue; BMI, body mass index; AST, aspartate transaminase; ALT, alanine transaminase; Hb, hemoglobin; Hct, hematocrit; WBC, white blood cell; LDL, low-density lipoprotein; HDL, high-density lipoprotein; ln, logarithmic transformation; Tg, triglyceride; HOMA-IR, homeostatic model assessment index-insulin resistance; CRP, c-reactive protein; AA, ascending aorta; SUV, standardized uptake value; HU, hounsfield unit.

### Comparisons between high and low VAT volume and VAT HU

Subjects were divided into 2 groups according to VAT volume and VAT HU. In high VAT volume (>3,298ml) and low VAT HU (≤-94.7) groups, both systolic and diastolic BP, BMI, waist circumference, insulin, lnHOMA-IR, CRP, AA SUV, lumbar SUV, and spleen SUV were higher. HDL cholesterol was lower in both high VAT volume (p = 0.0049) and low VAT HU (p = 0.0001) groups. Glucose (p = 0.0041), and liver SUV (p<0.0001) were higher in high VAT volume group, while LDL cholesterol (p = 0.0158) was higher in low VAT HU group.

### Univariable and multivariable regression analyses of VAT volume

In univariable analysis, VAT volume showed positive associations with BMI (coefficient 317.91, p<0.0001), waist circumference (144.58, p<0.0001), glucose (46.42, p = 0.0001), insulin (233.70, p = 0.0003), lnHOMA-IR (1228.11, p = 0.0002), and PET parameters of AA SUV (2218.06, p<0.0001), lumbar SUV (2175.40, p = 0.0001), liver SUV (1701.84, p = 0.0002), and spleen SUV (1973.62, p<0.0001) and negative associations with HDL cholesterol (-37.44, p = 0.0008), and VAT HU (-204.09, p<0.0001). In multivariable analysis, BMI (78.25, p = 0.0259), glucose (37.62, p<0.0001), insulin (348.90, p = 0.0011), lnHOMA-IR (-2118.37, p = 0.0007), and VAT HU (-134.99, p<0.0001) were independently associated with VAT volume. Table 2 shows the results of multivariable regression analyses of VAT volume.

**Table 2:** Multivariable Regression Analyses–VAT volume (adjustment with age and sex).

		Coefficient	t	p
	BMI	78.25	2.31	0.0259
Lab	HDL Cholesterol	-10.59	-1.83	0.0747
	Glucose	37.62	5.13	<0.0001
	Insulin	348.90	3.50	0.0011
	lnHOMA-IR	-2118.37	-3.63	0.0007
PET	AA SUV	-22.05	-0.08	0.9376
	Lumbar SUV	517.90	1.86	0.0701
	Liver SUV	205.72	0.84	0.4073
	Spleen SUV	77.86	0.30	0.7637
CT	VAT HU	-134.99	-7.95	<0.0001

VAT, visceral adipose tissue; BMI, body mass index; HDL, high-density lipoprotein; ln, logarithmic transformation; HOMA-IR, homeostatic model assessment index-insulin resistance; AA, ascending aorta; SUV, standardized uptake value; HU, hounsfield unit.

### Univariable and multivariable regression analyses of VAT HU

VAT HU was associated positively with HDL cholesterol (0.15, p = 0.0011) and negatively with BMI (-1.12, p<0.0001), waist circumference (-0.53, p<0.0001), total cholesterol (-0.04, p = 0.0346), LDL cholesterol (-0.05, p = 0.0104), lnTg (-4.08, p = 0.0040), glucose (-0.12, p = 0.0289), insulin (-0.88, p = 0.0013), lnHOMA-IR (-4.58, p = 0.0012), VAT volume (-0.004, p<0.0001), and PET parameters of AA SUV (-8.34, p = 0.0001), lumbar SUV (-5.65, p = 0.0192), liver SUV (-5.16, p = 0.0099), and spleen SUV (-6.20, p = 0.0023). In multivariable analysis, glucose (0.1187, p = 0.0098), and VAT volume (-0.004, p<0.0001) were found to be associated with VAT HU. Table 3 shows the results of multivariable regression analysis of VAT HU.

**Table 3:** Multivariable Regression Analyses–VAT HU (adjustment with age and sex).

		Coefficient	t	p
	BMI	0.15	0.68	0.5012
	Waist circumference	-0.08	-0.74	0.4630
Lab	Total cholesterol	-0.10	-1.62	0.1129
	LDL cholesterol	0.06	1.05	0.2996
	HDL cholesterol	0.06	0.91	0.3686
	lnTg	1.24	0.75	0.4595
	Glucose	0.12	2.71	0.0098
	Insulin	0.95	1.65	0.1059
	lnHOMA-IR	-6.46	-1.91	0.0634
PET	AA SUV	-2.08	-1.44	0.1580
	Lumbar SUV	2.40	1.61	0.1144
	Liver SUV	1.31	1.02	0.3144
	Spleen SUV	-0.42	-0.30	0.7678
CT	VAT volume	-0.004	-7.15	<0.0001

VAT, visceral adipose tissue; HU, hounsfield unit; BMI, body mass index; LDL, low-density lipoprotein; HDL, high-density lipoprotein; ln, logarithmic transformation; Tg, triglyceride; HOMA-IR, homeostatic model assessment index-insulin resistance; AA, ascending aorta; SUV, standardized uptake value.

## Discussion

It is well established that visceral adiposity is associated with cardiometabolic risk factors [17]. In this study, both VAT HU and VAT volume were significantly associated with cardiometabolic risk factors. In addition, VAT HU showed a strong association with VAT volume negatively. These results are similar with previous studies with VAT volume (table 4)[14, 15, 18–20]. CT scans were acquired as a substudy of the Framingham Heart Study[14, 15, 18, 19]. In all 4 reports from Framinghamg Heart Study, 25 contiguous 5-mm thickness CT scan was done in participants[14, 15, 18, 19]. Both volume and HU from VAT and SAT were measured from 25 contiguous 5-mm thickness CT scans. However, 25 contiguous 5-mm thickness CT covers 125 mm of abdomen above S1 level, which is a part of VAT in a subject [13]. In the other study by Tahara et al[20], they adopted PET/CT to measure the metabolic activity of fat tissue. However, 11 contiguous 4-mm thickness CT was included in measuring parameters, covering 44 mm of abdomen, a very small part of the whole abdomen. Until now, measurement of CT-based VAT area is common in a clinical setting. As VAT is distributed in whole abdomen, measuring an area on a single slice at L1 [21], L4/5 level[22, 23] or several slices from L2 to L5 [24] may not be enough to represent VAT of abdomen. Therefore, we measured a whole VAT delineating abdominal muscular wall, which might be the most accurate way to estimate VAT.

**Table 4:** Previous studies of VAT volume.

Author	Year	Image acquisition	Level	Variables	Parameters of fat
Rosenquist et al.[14]	2013	25 contiguous 5-mm thickness CT	above S1	VAT and SAT	Volume and HU
Britton et al.[18]	2013	25 contiguous 5-mm thickness CT	above S1	VAT and SAT	Volume
Alvey et al.[15]	2014	25 contiguous 5-mm thickness CT	above S1	VAT and SAT	Volume and HU
Tahara et al.[20]	2015	11 contiguous 4-mm thickness CT	umbilicus	VAT and SAT	Area, HU, and SUV
Rosenquist et al.[19]	2015	25 contiguous 5-mm thickness CT	above S1	VAT and SAT	Volume and HU

VAT, visceral adipose tissue; CT, computed tomography; SAT, subcutaneous adipose tissue; HU, hounsfield unit; SUV, standardized uptake value.

Interestingly, not only VAT volume, but also VAT HU were statistically associated with cardiometabolic risk factors in present study. The mechanisms are not fully clarified, however, a possible explanation is a role of adipocyte hypertrophy. Attenuation of tissue determined by CT is a marker of lipid content and decreased attenuation represents more lipid-dense fat tissue [25]. Cellular lipid contents help to determine adipocyte cell size [26]. Increased adipocyte cell size is associated with reduced number of preadipocytes which is a precursor cells able to differentiate into adipocyte [27]. Impaired adipocyte differentiation is well known to be related with cardiometabolic complications. Furthermore, increased adipocyte size is also correlated with decreased adiponectin concentrations [28]. It has been found that decreased plasma adiponectin concentrations are associated with progression of atherosclerosis and increased incidence of CVD[29]. Taken together, lower attenuation may represents adipocyte hypertrophy, which predicts more adverse cardiometabolic risk. It is certain that, increased amount of VAT is associated with adverse cardiometabolic risk [12]. Numerous studies have demonstrated that the VAT compartment is metabolically active and secreting multiple biologically active molecules such as adipokines, inflammatory markers, vasoactive substances, markers of hemostasis, and growth factors [12, 30, 31]. To assess amount of VAT, the gold standard is the volumetric CT measurement [5, 12]. Because CT is limited by amount of radiation exposure and high cost, waist circumference and BMI, the simple anthropometric markers, commonly used measurement for VAT in the clinical practice and there are data suggesting that theses indices are associated with cardiometabolic risk factors [32].

Although PET parameters of AA, lumbar spine, liver, and spleen were associated independently with neither VAT volume nor VAT HU, univariable analysis showed significant associations between PET parameters and both VAT volume and VAT HU. Recent studies reported that FDG uptake could be useful tool for the measurement of inflammation [33]. Histological studies indicated that FDG accumulation was localized within the atherosclerotic plaques [34]. Moreover, FDG uptake of aorta was significantly correlated with LDL-C concentration and atherosclerosis [35]. Tahara et al have presented that FDG uptake of VAT and SAT measured is involved in adipose tissue inflammation [20]. However, misregistration between PET and CT scans can be problematic to measure FDG activity in whole VAT and SAT. VAT and SAT show a faint FDG uptake in a normal subject with SUV less than 1 [36]. Including urine FDG activities from kidney, bladder, or ureter into VAT VOI can lead to increase in metabolic activity in VAT by the mistake. In addition, although VAT is delineated by abdominal muscular layer, SAT is not defined with upper or lower border. Therefore, measuring whole SAT can be subjective and problematic.

This study has several limitations. First, this is a cross sectional study with a small sample size with a retrospective design, thereby precluding inferences of causality or temporality. Second, although FDG-PER/CT imaging was performed and FDG uptake was assessed in the ascending aorta and spleen, we were unable to assess metabolic activity of VAT and SAT because of misregistration. Third, other non-invasive methods for cardiometabolic risks, such as calcium score or carotid intima-media thickness, could not be evaluated.

## Conclusion

Both VAT volume and VAT HU is significantly associated with cardiometabolic risk factors. This is the first study that measured whole abdominal cavity to estimate VAT.

## Supporting Information

S1 File Data of this study. (ZIP)

## References

[1] Prevention KCfDCa. 2013 The Korea National Health and Nutrition Examination Survey.

[2] Joshy G, Korda RJ, Attia J, Liu B, Bauman AE, Banks E. Body mass index and incident hospitalisation for cardiovascular disease in 158 546 participants from the 45 and Up Study. Int J Obes (Lond). 2014;38(6):848–56. 10.1038/ijo.2013.192 24149770 PMC4052432

[3] Visscher TL, Rissanen A, Seidell JC, Heliovaara M, Knekt P, Reunanen A, et al Obesity and unhealthy life-years in adult Finns: an empirical approach. Arch Intern Med. 2004;164(13):1413–20. 10.1001/archinte.164.13.1413 . 15249350

[4] Finucane MM, Stevens GA, Cowan MJ, Danaei G, Lin JK, Paciorek CJ, et al National, regional, and global trends in body-mass index since 1980: systematic analysis of health examination surveys and epidemiological studies with 960 country-years and 9.1 million participants. Lancet. 2011;377(9765):557–67. 10.1016/S0140-6736(10)62037-5 21295846 PMC4472365

[5] Fox CS, Massaro JM, Hoffmann U, Pou KM, Maurovich-Horvat P, Liu CY, et al Abdominal visceral and subcutaneous adipose tissue compartments: association with metabolic risk factors in the Framingham Heart Study. Circulation. 2007;116(1):39–48. 10.1161/CIRCULATIONAHA.106.675355 . 17576866

[6] Rosito GA, Massaro JM, Hoffmann U, Ruberg FL, Mahabadi AA, Vasan RS, et al Pericardial fat, visceral abdominal fat, cardiovascular disease risk factors, and vascular calcification in a community-based sample: the Framingham Heart Study. Circulation. 2008;117(5):605–13. 10.1161/CIRCULATIONAHA.107.743062 . 18212276

[7] Irlbeck T, Massaro JM, Bamberg F, O'Donnell CJ, Hoffmann U, Fox CS. Association between single-slice measurements of visceral and abdominal subcutaneous adipose tissue with volumetric measurements: the Framingham Heart Study. Int J Obes (Lond). 2010;34(4):781–7. 10.1038/ijo.2009.279 20065971 PMC2982778

[8] Han JH, Park HS, Kim SM, Lee SY, Kim DJ, Choi WH. Visceral adipose tissue as a predictor for metabolic risk factors in the Korean population. Diabet Med. 2008;25(1):106–10. 10.1111/j.1464-5491.2007.02317.x . 18028439

[9] Demerath EW, Sun SS, Rogers N, Lee M, Reed D, Choh AC, et al Anatomical patterning of visceral adipose tissue: race, sex, and age variation. Obesity (Silver Spring). 2007;15(12):2984–93. 10.1038/oby.2007.356 18198307 PMC2883307

[10] Greenfield JR, Samaras K, Chisholm DJ, Campbell LV. Regional intra-subject variability in abdominal adiposity limits usefulness of computed tomography. Obes Res. 2002;10(4):260–5. 10.1038/oby.2002.35 . 11943834

[11] Shen W, Punyanitya M, Chen J, Gallagher D, Albu J, Pi-Sunyer X, et al Visceral adipose tissue: relationships between single slice areas at different locations and obesity-related health risks. Int J Obes (Lond). 2007;31(5):763–9. 10.1038/sj.ijo.0803474 17060927 PMC3166348

[12] Wajchenberg BL. Subcutaneous and visceral adipose tissue: their relation to the metabolic syndrome. Endocr Rev. 2000;21(6):697–738. 10.1210/edrv.21.6.0415 . 11133069

[13] Maurovich-Horvat P, Massaro J, Fox CS, Moselewski F, O'Donnell CJ, Hoffmann U. Comparison of anthropometric, area- and volume-based assessment of abdominal subcutaneous and visceral adipose tissue volumes using multi-detector computed tomography. Int J Obes (Lond). 2007;31(3):500–6. 10.1038/sj.ijo.0803454 . 16953256

[14] Rosenquist KJ, Pedley A, Massaro JM, Therkelsen KE, Murabito JM, Hoffmann U, et al Visceral and subcutaneous fat quality and cardiometabolic risk. JACC Cardiovasc Imaging. 2013;6(7):762–71. 10.1016/j.jcmg.2012.11.021 23664720 PMC3745280

[15] Alvey NJ, Pedley A, Rosenquist KJ, Massaro JM, O'Donnell CJ, Hoffmann U, et al Association of fat density with subclinical atherosclerosis. Journal of the American Heart Association. 2014;3(4). 10.1161/JAHA.114.000788 25169793 PMC4310364

[16] Matthews DR, Hosker JP, Rudenski AS, Naylor BA, Treacher DF, Turner RC. Homeostasis model assessment: insulin resistance and beta-cell function from fasting plasma glucose and insulin concentrations in man. Diabetologia. 1985;28(7):412–9. . 3899825 10.1007/BF00280883

[17] Shah RV, Murthy VL, Abbasi SA, Blankstein R, Kwong RY, Goldfine AB, et al Visceral adiposity and the risk of metabolic syndrome across body mass index: the MESA Study. JACC Cardiovasc Imaging. 2014;7(12):1221–35. 10.1016/j.jcmg.2014.07.017 25440591 PMC4268163

[18] Britton KA, Massaro JM, Murabito JM, Kreger BE, Hoffmann U, Fox CS. Body fat distribution, incident cardiovascular disease, cancer, and all-cause mortality. Journal of the American College of Cardiology. 2013;62(10):921–5. 10.1016/j.jacc.2013.06.027 23850922 PMC4142485

[19] Rosenquist KJ, Massaro JM, Pedley A, Long MT, Kreger BE, Vasan RS, et al Fat quality and incident cardiovascular disease, all-cause mortality, and cancer mortality. The Journal of clinical endocrinology and metabolism. 2015;100(1):227–34. 10.1210/jc.2013-4296 . 25226289 PMC5399496

[20] Tahara N, Yamagishi S, Kodama N, Tahara A, Honda A, Nitta Y, et al Clinical and biochemical factors associated with area and metabolic activity in the visceral and subcutaneous adipose tissues by FDG-PET/CT. The Journal of clinical endocrinology and metabolism. 2015;100(5):E739–47. 10.1210/jc.2014-3896 . 25695885

[21] Kim D, Chung GE, Kwak MS, Seo HB, Kang JH, Kim W, et al Body Fat Distribution and Risk of Incident and Regressed Nonalcoholic Fatty Liver Disease. Clinical gastroenterology and hepatology: the official clinical practice journal of the American Gastroenterological Association. 2015 10.1016/j.cgh.2015.07.024 . 26226099

[22] Murphy RA, Reinders I, Register TC, Ayonayon HN, Newman AB, Satterfield S, et al Associations of BMI and adipose tissue area and density with incident mobility limitation and poor performance in older adults. The American journal of clinical nutrition. 2014;99(5):1059–65. 10.3945/ajcn.113.080796 24522448 PMC3985211

[23] Nakamura K, Hongo A, Kodama J, Hiramatsu Y. Fat accumulation in adipose tissues as a risk factor for the development of endometrial cancer. Oncology reports. 2011;26(1):65–71. 10.3892/or.2011.1259 . 21491090

[24] Abbasi SA, Hundley WG, Bluemke DA, Jerosch-Herold M, Blankstein R, Petersen SE, et al Visceral adiposity and left ventricular remodeling: The Multi-Ethnic Study of Atherosclerosis. Nutr Metab Cardiovasc Dis. 2015;25(7):667–76. 10.1016/j.numecd.2015.03.016 26033394 PMC4468023

[25] Baba S, Jacene HA, Engles JM, Honda H, Wahl RL. CT Hounsfield units of brown adipose tissue increase with activation: preclinical and clinical studies. J Nucl Med. 2010;51(2):246–50. 10.2967/jnumed.109.068775 . 20124047

[26] Wronska A, Kmiec Z. Structural and biochemical characteristics of various white adipose tissue depots. Acta Physiol (Oxf). 2012;205(2):194–208. 10.1111/j.1748-1716.2012.02409.x . 22226221

[27] Isakson P, Hammarstedt A, Gustafson B, Smith U. Impaired preadipocyte differentiation in human abdominal obesity: role of Wnt, tumor necrosis factor-alpha, and inflammation. Diabetes. 2009;58(7):1550–7. 10.2337/db08-1770 19351711 PMC2699851

[28] Henninger AM, Eliasson B, Jenndahl LE, Hammarstedt A. Adipocyte hypertrophy, inflammation and fibrosis characterize subcutaneous adipose tissue of healthy, non-obese subjects predisposed to type 2 diabetes. PLoS One. 2014;9(8):e105262 10.1371/journal.pone.0105262 25148116 PMC4141784

[29] Lopez-Jaramillo P, Gomez-Arbelaez D, Lopez-Lopez J, Lopez-Lopez C, Martinez-Ortega J, Gomez-Rodriguez A, et al The role of leptin/adiponectin ratio in metabolic syndrome and diabetes. Horm Mol Biol Clin Investig. 2014;18(1):37–45. 10.1515/hmbci-2013-0053 . 25389999

[30] Kanaya AM, Harris T, Goodpaster BH, Tylavsky F, Cummings SR, Health A, et al Adipocytokines attenuate the association between visceral adiposity and diabetes in older adults. Diabetes Care. 2004;27(6):1375–80. . 15161791 10.2337/diacare.27.6.1375

[31] Saijo Y, Kiyota N, Kawasaki Y, Miyazaki Y, Kashimura J, Fukuda M, et al Relationship between C-reactive protein and visceral adipose tissue in healthy Japanese subjects. Diabetes Obes Metab. 2004;6(4):249–58. 10.1111/j.1462-8902.2003.0342.x . 15171748

[32] Balkau B, Deanfield JE, Despres JP, Bassand JP, Fox KA, Smith SC Jr., et al International Day for the Evaluation of Abdominal Obesity (IDEA): a study of waist circumference, cardiovascular disease, and diabetes mellitus in 168,000 primary care patients in 63 countries. Circulation. 2007;116(17):1942–51. 10.1161/CIRCULATIONAHA.106.676379 17965405 PMC2475527

[33] Zhuang H, Alavi A. 18-fluorodeoxyglucose positron emission tomographic imaging in the detection and monitoring of infection and inflammation. Semin Nucl Med. 2002;32(1):47–59. 10.1053/snuc.2002.29278 . 11839069

[34] Rudd JH, Warburton EA, Fryer TD, Jones HA, Clark JC, Antoun N, et al Imaging atherosclerotic plaque inflammation with [18F]-fluorodeoxyglucose positron emission tomography. Circulation. 2002;105(23):2708–11. . 12057982 10.1161/01.cir.0000020548.60110.76

[35] Haraguchi A, Hayashida N, Kamasaki T, Miyamoto I, Usui T, Ando T, et al Uptake of aortic 18F-FDG is correlated with low-density lipoprotein cholesterol and leptin in a general population. PLoS One. 2014;9(11):e111990 10.1371/journal.pone.0111990 25375161 PMC4222970

[36] Heusch P, Buchbender C, Beiderwellen K, Nensa F, Hartung-Knemeyer V, Lauenstein TC, et al Standardized uptake values for [(1)(8)F] FDG in normal organ tissues: comparison of whole-body PET/CT and PET/MRI. European journal of radiology. 2013;82(5):870–6. 10.1016/j.ejrad.2013.01.008 . 23394765

